# Short Report: TRPV1-polymorphism 1911 A>G alters capsaicin-induced sensory changes in healthy subjects

**DOI:** 10.1371/journal.pone.0183322

**Published:** 2017-08-17

**Authors:** Julia Forstenpointner, Matti Förster, Denisa May, Friederike Hofschulte, Ingolf Cascorbi, Gunnar Wasner, Janne Gierthmühlen, Ralf Baron

**Affiliations:** 1 Division of Neurological Pain Research and Therapy, Department of Neurology, University Hospital Schleswig-Holstein, Campus Kiel, Kiel, Germany; 2 Department of Neurology, Neurophysiology, Neuropsychology and Stroke Unit, Bogenhausen Hospital STKM, Munich, Germany; 3 Institute of Experimental and Clinical Pharmacology, University Hospital Schleswig-Holstein, Campus Kiel, Kiel Germany; 4 Neurological Clinic, Kiel, Germany; University of Würzburg, GERMANY

## Abstract

**Background:**

C-fibers express transient receptor potential (TRP) channels. These high-voltage gated channels function as integrators of different physical stresses (e.g. heat, protons, ATP). Additionally channel activation can be induced by capsaicin. Topically applied, capsaicin elicits burning pain, heat and mechanical hyperalgesia and serves as a human surrogate model for pain. It was suggested that the TRPV1-variant rs8065080 (1911A>G) plays a pivotal role in patients with neuropathic pain syndromes. We investigated the effect of this TRPV1-SNP on thermal sensitivity and superficial skin perfusion in 25 healthy subjects.

**Methods and findings:**

Nine subjects being homozygous TRPV1 wild type (AA), 8 heterozygous (AG) and 8 homozygous variant (GG) carriers were selected out of a pool of genotyped healthy individuals. Under physiological conditions (no capsaicin application), there was no statistical significant difference in thermal thresholds or skin perfusion between carriers of different TRPV1 1199A>G genotypes. However, intra-individual calculations (Δ% pre vs. post capsaicin) revealed (1) less warm-detection in AA/AG (-82.1%) compared to GG (-13.1%) and (2) a gain of heat pain sensitivity in AA/AG (+22.2%) compared to GG carriers (+15.6%) after adjustment for perfusion measurements ((1)p = 0.009, (2)p = 0.021).

**Conclusion:**

Presence of homozygous variant TRPV1 genotype (GG) demonstrated less capsaicin-induced warm hypoesthesia in warm-detection and less capsaicin-induced heat pain sensitivity suggesting an altered channel function. This demonstrates not only the functional influence of TRPV1 rs8065080 polymorphism itself; it further more underpins the relevance of genotyping-based approaches in both patients and surrogate models of neuropathic pain in healthy volunteers.

## Introduction

Transient receptor potential (TRP) channels are high-threshold voltage-gated cation channels expressed on small diameter afferent nerves. They serve as integrators of different physical stressors transducing these into electrical impulses [1]. TRP vanilloid (V) 1 receptor became of interest for pain research, when Caterina et al. described its cloning and the channel’s influence on heat pain thresholds [2, 3]. Among other stimuli (e.g. H^+^, ATP, heat) TRPV1 can be activated by capsaicin inducing a burning pain sensation [1]. Recently, several findings suggested that TRPV1 plays a role in neuropathic pain syndromes [4]. These are defined as chronic pain directly caused by a lesion or disease of the somatosensory system [5]. Protein expression analysis showed an increase of TRPV1 in damaged nerve fibers and corresponding dorsal root ganglia (DRG) and moreover in adjacent non-damaged afferents of partially damaged nerves [6–8].

Several human surrogate models mimicking neuropathic pain symptoms are available to investigate underlying pathophysiological mechanisms [9]. The capsaicin model, using the ingredient of capsicum peppers as stimulus is well established [10]. Topical application of capsaicin (in low concentrations (0.6%)) on the skin of human volunteers induces burning pain sensations and heat hyperalgesia in the area of application [11]. Extending this primary zone, a second area exhibiting lower thresholds to punctate and dynamic mechanical stimuli can be specified (secondary area). Besides these evoked symptoms, a visible erythema (flare) caused by axon reflex activation of C-fibers appears [12].

There are several lines of evidence that pain perception and sensation varies intra-individually. Many patients with the same etiology and natural history of their disease experience pain differently. It was suggested that different mechanisms are responsible for this high variability [13, 14]. The causes are not entirely clear but the influence of genetic factors becomes more evident [15, 16]. TRPV1 has been identified as a potential target for pain therapy as it plays a key role in nociceptive signaling, but clinical studies with channel antagonists have struggled with side effects [17–19]. Moreover, during topical application of a high dose (8%) capsaicin patch, the acute evoked pain intensity as well as the therapeutic response is individually variable [20]. Hence, the question arises whether TRPV1 variants influence pain perception.

Among the rs8065080 (1911A>G) variant, which we explore in the presented study other single nucleotide polymorphisms (SNPs) have been identified to play a role in human pain perception. The rs222747 (1103C>G) variant for instance was associated with cold hypaesthesia, this however might be the result of a functional interaction between the TRPM8 and the TRPV1 receptor, inducing a SNP controlled inhibition of the TRPM8 [4, 21, 22]. Further the rs222741 (2841C>T) variant was associated with migraine in a large cohort of patients [23]. Beside this very evident findings of altered pain perception in different TRPV1 polymorphisms, the current study concentrates on the 1911A>G variant. This attempt was supported by a line of contrary explorations, for instance two *in vitro* studies explored TRPV1 1911A>G in oocytes and HEK293 cells suggesting that the substitution does not alter receptor function [24, 25]. In contrast Kim et al. found that cold withdrawal time in men is impaired by this SNP indicating impaired thermal pain sensitivity [15]. *In vitro* investigations in HeLa cells revealed a decreased channel function by the variant 585V protein [26]. Previously, the distribution frequency of single nucleotide polymorphisms (SNP) in the TRPV1 gene in large cohorts of neuropathic pain patients was defined [4]. It was shown that neuropathic pain patients with preserved sensory function carrying TRPV1 1911A<G variants (I585V, rs8065080) were protected to develop heat hyperalgesia. However, the study did not confirm *TRPV1* as a general pain susceptibility gene. Despite these findings in neuropathic pain patients the functional consequence of TRPV1 1911A>G, in healthy subjects, remains to be investigated. On the basis of these previous findings, we hypothesize that the TRPV1 variant 1911A>G may influence physiological channel function, e.g. thermal sensitivity and superficial skin perfusion in the human capsaicin pain model.

Whether this is caused by the variant gene product itself or a disease-specific gene-environment-interaction remains unsolved.

The investigation of functional *in vivo* consequences of the non-synonymous SNP 1911A>G in the TRPV1 gene will help to answer this important question.

## Materials and methods

25 volunteers (6 male, 19 female), mean age 23.8 (± 0.39) years, range 22–29 years, participated in this study after having signed informed consent prior to the study (S1 and S2 Figs). The Ethics Committee of the Medical Faculty of the Christian Albrechts University of Kiel approved the study. All protocols conformed to guidelines as set by the Declaration of Helsinki.

All 25 subjects were selected between 8/2011-1/2012 according to the TRPV1 1911G>A genotype out of a cohort of 350 healthy individuals, who had been recruited by the Institute of Experimental and Clinical Pharmacology from 2009 until 2012 and are registered in the “popgen 2.0” biobank. Out of 38 subjects who were approached, 25 volunteers agreed to take part and all finished the study. Participants were free of cardiovascular, renal, gastrointestinal, pulmonary, dermatological and neurological disorders determined by medical history and physical examination [4]. They did not take any medications aside from contraceptives.

Whole genomic DNA (gDNA) was extracted from venous blood samples using the Qiagen Gentra Puregene Blood Kit (Qiagen, Hilden, Germany). The final concentration of gDNA was estimated spectrophotometrically. Genotyping of TRPV1 1911A>G (rs8065080) was performed by pyrosequencing according to protocols provided by the manufacturer on a PSQ HS96 platform (Biotage AB, Uppsala, Sweden). Primer sequences and conditions were described previously [4].

The phenotypical characterization was performed before, continuously and after the application of a capsaicin solution (0.6% in 40% ethanol) patch (3x3cm gauze pad) at the right medial forearm. The patch was removed after 15 minutes. Capsaicin application has been described elsewhere in detail [11].

**Fig 1:**
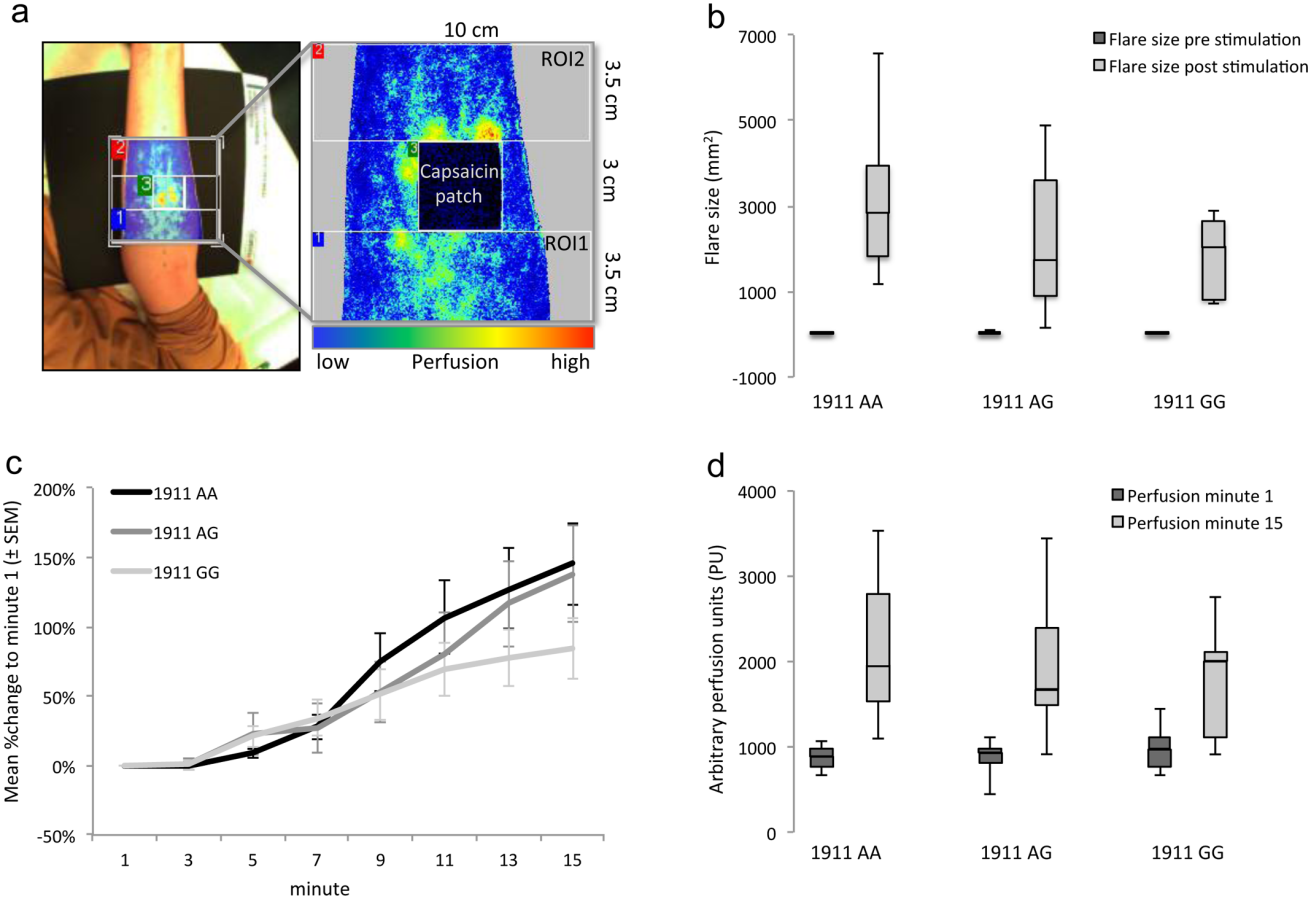
Laser Doppler measurement. **a)** Sample image of Laser Doppler measurement setup. Regions of interest (ROI) were created around the capsaicin patch stimulation area for further calculations. The intensity image depicts the color-coded perfusion magnitude. **b)** Area of elevated skin perfusion (flare) was measured in mm^2^. There was no significant difference between the genotypes, however a trend towards smaller flare areas in 1911 GG carriers can be observed. **c-d**) Perfusion changes during capsaicin application were measured via Laser Doppler flowmetry. There was no significant difference between the genotypes, but a trend towards a decreased perfusion gain in 1911 GG carriers.

A (1) perfusion measurement system (PeriCam PSI System, 785nm, Perimed, Sweden) using real-time laser speckle contrast analysis determined the flare area and the perfusion in a field of 10x10 cm (resolution 0.18 mm, 3 frames per second) with the laser Doppler probe locked 20 cm above the forearm. The flare area was automatically calculated in mm^2^ from pixels of the entire picture that exceeded baseline values by two standard deviations. Perfusion needed to be determined in an area surrounding the gauze pad as this prevented measurements within the stimulation (primary) area. For this purpose two regions of interest (ROI) proximal and distal to the pad as provided by the software (PimSoft, Perimed, Sweden) were created (Fig 1). Relative perfusion units expressed perfusion and the area under the curve (AUC) was calculated for a timeframe of five seconds.

Secondly (2) QST i.e. thermal quantitative sensory testing was conducted according to the protocol of the German research Network on Neuropathic Pain (DFNS) (Rolke et al. 2006). The small fiber function in the stimulated area was assessed by a shortened standardized assessment containing 4 different thermal tests: thermal detection thresholds for the perception of cold (CDT: cold detection threshold) and warmth (WDT: warm detection threshold), and thermal pain thresholds for cold (CPT: cold pain threshold) and heat (HPT: heat pain threshold). The testing procedure was performed by a thermotest-device (TSA, medoc, Israel), with a 3x3cm thermo probe placed at the volar forearm.

Skin and room temperature were kept at a constant level (mean room temperature: 24.2°C; range: 23–26°C) during the entire experimentation period and did not differ between the groups.

### Statistical analysis

Data is displayed as mean (± standard error) or range. A Shapiro-Wilk test was used to test for normal distribution. Group differences before and after capsaicin application as well as intra-individual changes of measurements (Δ%change prior vs. after capsaicin) were calculated.

First group comparisons of TRPV1 1911 AA/AG/GG carriers were calculated by a one-way ANOVA on ranks, post hoc a Mann Whitney U test was conducted. Additionally to compare AA/AG to homozygous GG variant carriers, wild type and heterozygous AG variant carriers were clustered.

Furthermore an ANCOVA or an ANCOVA on rank transformed data was performed to correct for covariance (perfusion) where appropriate. The mentioned statistical approach was selected in accordance to Conover et al. and Vickers et al. [27], [28]. Correlation analysis was conducted via Pearson’s rank correlation analysis. Bonferroni-Holm correction for multiple comparisons was applied. Differences at p<0.05 were considered as significant. Statistical analysis was performed using SPSS (IBM USA), version 22.

## Results

A difference in time to onset of pain after capsaicin application could be detected between AA/AG and GG carriers. A Mann-Whitney U test revealed a statistical significant difference (p = 0.009) between AA/AG 5.59min (±2.62) and GG 2.50min (±2.33).

Thermal quantitative testing revealed an intra-individual group difference (p = 0.029) in warm detection thresholds (WDT) towards loss of warm detection in TRPV1 1911AA (-92.6%)/AG (-70.4%) compared to homozygous variant GG carriers (-13.1%). Post hoc a Mann-Whitney U test revealed a significant group difference between AA and GG carriers by p = 0.008.

Other than that, data of the thermal quantitative sensory testing parameters CDT, CPT and HPT did not reach any significance level after multiple comparisons.

**Fig 2:**
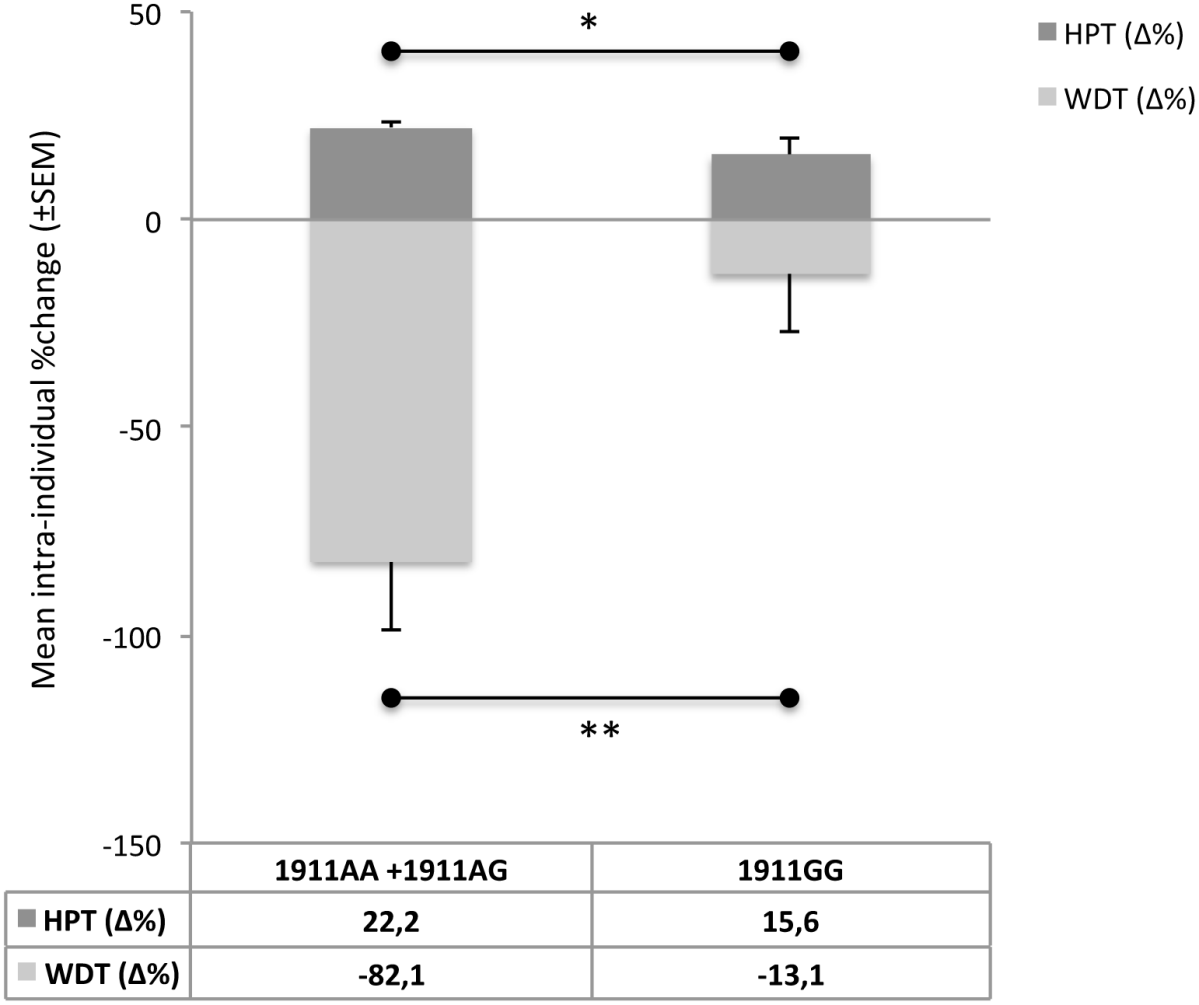
Thermal quantitative sensory testing of TRPV1 variants. Comparison of intra-individual data (Δ%change prior vs. after capsaicin) of homozygous G (1911GG) against homozygous and heterozygous A carriers (1911AA+1911AG) reveals lower changes (1) towards loss of warm detection in GG (13.1%) compared to AA/AG (82.1%) and (2) towards gain of heat pain sensitivity in GG (15.6%) compared to AA/AG (22.2%) ((1) p = 0.009; (2) p = 0.021). Note: Data are displayed as means (±SEM) for all analyzed patients. P<0.05 considered significance. ((1)Mann-Whitney U test, (2)ANCOVA). *p<0.05; **p<0.01.

However after correction for perfusion measurements (AUC) and after clustering (AA/AG vs. GG) intra-individual data (Δ%change), comparison of heat pain thresholds (HPT) reached significance of p = 0.021, i.e. indicating a gain of heat pain sensitivity in AA/AG (+22.2%) compared to GG (+15.6%) (Fig 2).

In all variant carriers perfusion and flare measurements were steadily increasing in the patch-surrounding skin after capsaicin application (Fig 1). A statistical difference between the groups prior or after capsaicin application as well as intra-individual data (Δ%change) comparisons did not detect any statistical significance. However after 15 minutes of capsaicin application, perfusion in AA wild types had increased by 145% and in heterozygote AG variants by 137%, while homozygote GG variants had doubled their superficial perfusion (84%) (Fig 1).

It is notable that TRPV1 1911GG variants exposed the smallest flare area (1830 mm^2^ (± 340)) while TRPV1 1911AG and AA had consecutively larger areas (2270 mm^2^ (± 630); 3110 mm^2^ (± 630)), though not reaching significance level (Fig 1).

Furthermore a Pearson’s rank correlation analysis revealed a strong correlation of intra-individual changes in WDT and flare (p = 0.009, r = 0.522).

## Discussion

Complementary to clinical association studies in neuropathic pain [4] and painful knee osteoarthritis [29], the presented study investigated the consequences of TRPV1 1911 A>G on somatosensory and perfusion function in normal and capsaicin challenged skin of healthy subjects. Our experiments revealed three main findings: (1) There are no differences between TRPV1 wild type and variant carriers under non-stimulated (i.e. physiological) conditions. (2) Capsaicin stimulation induces less warm hypoesthesia and less heat pain sensitivity in TRPV1 variant carriers. Thus, our results are not in line with preclinical *in vitro* findings revealing that the variant does not have an influence on channel function in response to heat or capsaicin [24, 25]. In contrast, there are several reports that the SNP TRPV1 1103C>G shows an increased capsaicin-initiated channel activity and a higher expression of the protein [25]. Also, Binder et al. found differences in heat pain thresholds in a large cohort of neuropathic pain patients carrying TRPV1 1911A>G variants [4]. These results are in line to our findings.

(3) Smaller areas of vasodilation and milder increase in superficial skin perfusion in homozygous TRPV1 1911GG carriers were observed (although not significant). It is thought that antidromic excitation of capsaicin-activated C-fiber afferents originating in the primary stimulated area elicits the release of neuropeptides (neurogenic inflammation, axon reflex), e.g. CGRP [12]. CGRP dilates arterioles resulting in an increased perfusion (flare) even outside the primary area due to branches from axon collaterals. Our results showed homozygous variant carriers developing smaller perfusion increment from baseline, which did not reach the magnitude of wild type or heterozygote variant carriers. Also, the total size of flares developed accordingly. This may support the TRPV1 1911A>G dependent influence on peripheral C-fiber sensitization.

The presented data indicate functional differences in the 1911A/G variants. These sensory and adaptive (see time to onset of pain) changes in the TRPV1 polymorphism might be the result of altered channel morphology. Moreover a capsaicin dependent reversible change of channel conformation might also be a possible explanation. The functional aspect however suggests a rather reversible effect due to the fact that no sensory change could be detected prior or after capsaicin application. Consequently the authors would expect that a shifted expression of the TRPV1 channel itself would lead to a different sensory perception initially (prior capsaicin application).

Constant stimulation of the TRPV1 channel potentially leads to an adjustment of the channel’s conformation/morphology e.g. in neuropathic pain patients. This hypothesis might be the foundation of the sensory alterations demonstrated by Binder et al. Besides also ageing processes could change the channels plasticity. These speculations however need to be confirmed elsewhere.

The presented evidence, demonstrating alterations in 1911A/G variants, probably reflect an altered channel morphology or a stimulus dependent change of channel conformation. Though further investigations on TRPV1-variant rs8065080 need to confirm the proposed mechanisms in a morphological based exploration.

## Limitations

The testing procedure was conducted at the volar forearm this might be seen as a limitation due to the fact that the reference data set adheres to the hand. Recently this became evident in a publication by Dimova et al. (2017), who demonstrate sensory differences in adjacent body areas [30]. Consequently this leads to the decision not to conduct a z-transformation, which would correct for testing area, age and gender.

Further the authors regret not to obtain any data from skin punch biopsies, which could have contributed valuably to the findings.

## Supporting information

S1 File (XLSX)

S2 File (XLSX)
